# Differences and similarities between IgG4-related disease with and without dacryoadenitis and sialoadenitis: clinical manifestations and treatment efficacy

**DOI:** 10.1186/s13075-019-1828-8

**Published:** 2019-02-01

**Authors:** Mu Wang, Panpan Zhang, Wei Lin, Yunyun Fei, Hua Chen, Jing Li, Li Zhang, Wenjie Zheng, Yongze Li, Xiaofeng Zeng, Jiaxin Zhou, Yamin Lai, Xiaowei Liu, Huadan Xue, Yueying Cui, Lian Zhou, Jizhi Zhao, Wen Zhang

**Affiliations:** 1 Department of Stomatology, Peking Union Medical College Hospital, Chinese Academy of Medical Sciences & Peking Union Medical College, No.1 Shuaifuyuan, Wangfujing, Dongcheng District, Beijing, 100730 China; 2 Department of Rheumatology, Peking Union Medical College Hospital, Chinese Academy of Medical Sciences & Peking Union Medical College, Key Laboratory of Rheumatology and Clinical Immunology, Ministry of Education, No.1 Shuaifuyuan, Wangfujing, Dongcheng District, Beijing, 100730 China; 3 Department of Rheumatology, Hei Bei Ren Min Hospital, No.1 Shuaifuyuan, Wangfujing, Dongcheng District, Beijing, 100730 China; 4 Department of Gastroenterology, Peking Union Medical College Hospital, Chinese Academy of Medical Sciences & Peking Union Medical College, No.1 Shuaifuyuan, Wangfujing, Dongcheng District, Beijing, 100730 China; 5 Department of ophthalmology, Peking Union Medical College Hospital, Chinese Academy of Medical Sciences & Peking Union Medical College, No.1 Shuaifuyuan, Wangfujing, Dongcheng District, Beijing, 100730 China; 6 Department of Radiology, Peking Union Medical College Hospital, Chinese Academy of Medical Sciences & Peking Union Medical College, No.1 Shuaifuyuan, Wangfujing, Dongcheng District, Beijing, 100730 China; 7 Institute of medical information, Chinese academy of medical sciences, Yabao Road 5th, Chaoyang District, Beijing, 100020 China

**Keywords:** IgG4-RD, Dacryoadenitis, Sialoadenitis, IgG4-RD RI, Treatment

## Abstract

**Background:**

This study aimed to compare the differences and similarities in the clinical manifestations and treatment efficacy of IgG4-related disease (IgG4-RD) in patients with and without dacryoadenitis and sialoadenitis (DS).

**Methods:**

A total of 121 untreated IgG4-RD patients in Peking Union Medical College Hospital were enrolled in this study. The patients were divided into three groups: DS-predominant (group A), non-DS (group B), and DS with other internal organs affected (group C). The patients were followed up for at least 15 months. Baseline and follow-up data were collected. The disease activity was evaluated according to the IgG4-RD responder index.

**Results:**

The mean ± SD age at disease onset was 53.2 ± 14.1 years, and 71.9% of the patients were male. The prevalence of allergies was higher in groups A (21, 61.8%) and C (32, 69.6%) than group B (14, 34.1%). More patients with DS (17, 50.0%, and 17, 37.0%) had sinonasal lesions than those without DS (5, 12.2%). Moreover, an increased number of eosinophils were more common in patients with DS than in those without, as were increased serum IgG, IgG4, and IgE levels. More patients in group B and group C (28, 68.3%, and 31, 67.4%) received a combination therapy of corticosteroid and immunosuppressant. During the 15-month follow-up, 28 (23.1%) patients had disease relapse.

**Conclusion:**

Results demonstrated that IgG4-RD patients with DS had distinctive clinical features compared with non-DS. Allergy and sinonasal involvement were more common in patients with DS. Patients with DS showed higher serum IgG4 levels than those without DS.

**Electronic supplementary material:**

The online version of this article (10.1186/s13075-019-1828-8) contains supplementary material, which is available to authorized users.

## Introduction

IgG4-related disease (IgG4-RD) is a fibro-inflammatory disease with single or multi-organ involvement; the pathology of the involved tissue includes a large amount of lymphocyte and plasma cell infiltration, storiform fibrosis, and obliterative phlebitis, accompanied by eosinophilia of the related tissue [1–3]. The clinical presentation of IgG4-RD is usually indolent and mostly affects the submandibular and lacrimal glands as well as the pancreas [3–5].

IgG4-RD is a highly heterogeneous entity that can affect various organs. Dacryoadenitis and sialoadenitis (DS) is the bilateral, painless, and symmetrical swelling of the lacrimal and salivary glands, previously referred to as “Mikulicz’s disease” by some researchers [2, 6–8]. Many of these patients were found to have elevated serum IgG4 levels and infiltration of IgG4-expressing plasma cells in the lacrimal and salivary glands and are recognized to have IgG4-RD [9–11], defined in this clinical presentation as IgG4-related dacryoadenitis and sialoadenitis (DS) [2, 12–14]. IgG4-related DS has been recognized as one of the most common presentations of IgG4-RD [4, 5, 9, 15, 16]. IgG4-DS can combine with other organ involvement, such as autoimmune pancreatitis, retroperitoneal fibrosis, tubulointerstitial nephritis, and lung disease [9]. Kubota et.al reported a small cohort in AIP patients with or without Mikulicz’s disease (MD), but only five MD patients were included [17]. So far, there has been no larger prospective cohort study to explore the onset symptoms, laboratory parameters, or treatment efficacy in IgG4-RD patients with and without DS. To further understand these distinctions, we analysed the clinical presentation, laboratory examination results, and treatment efficacy in patients with and without DS.

## Methods

### Patients

In this study in Peking Union Medical College Hospital, 121 untreated patients with IgG4-RD who fulfilled the 2011 comprehensive IgG4-RD diagnostic criteria were prospectively enrolled from January 2011 to January 2016 [18]. The diagnosis of IgG4-RD was based on the following criteria: (1) a clinical examination showing characteristic diffuse/localized swelling or masses in single or multiple organs, (2) an elevated serum IgG4 concentration (> 135 mg/dL), and (3) a histopathologic examination showing (a) marked lymphocytic and plasma cell infiltration and fibrosis or (b) the infiltration of IgG4+ plasma cells (a ratio of IgG4+/IgG+ cells > 40% and > 10 IgG4+ plasma cells per high power field). Patients with cancer, lymphoma, or other autoimmune diseases were excluded. According to the affected anatomic sites by clinical symptoms, physical examinations and imaging, including ultrasound scanning, computed tomography (CT), or magnetic resonance imaging (MRI), or positron emission tomography/computed tomography (PET/CT), the patients were divided into three groups: group A, DS-predominant (patients with lymph node and nasal/sinus involvements could be included); group B, other affected organs but without DS; and group C, dacryoadenitis and/or sialoadenitis and other internal organs affected. History of allergy was collected by using the criteria from the European Academy of Allergy and Clinical Immunology. The patients were followed up at 1 month, 3 months, and then approximately every 3 months for at least 15 months in total. Blood and essential imaging examinations were conducted, and the IgG4-RD responder index (IgG4-RD RI, 2015 version) was evaluated at each follow-up [19, 20] (Additional file 1). A relapse was defined by a new development or the return of abnormal findings upon physical examination, in laboratory tests reflecting IgG4-RD activity within specific organs, or on imaging studies [21, 22]. An increase of more than 2 in a patient’s IgG4-RD RI was considered a disease relapse. This study was approved by the ethics committee, and written informed consents were obtained.

### Clinical data and laboratory parameters

The clinical data included age, sex, disease duration, treatment, allergy history, symptoms at disease onset, number of follow-up months, and the IgG4-RD RI at baseline and at each follow-up examination. Laboratory parameters included routine blood analysis; liver function; kidney function; serum immunoglobulin G, A, and M; serum IgG subclass; serum IgE; anti-nuclear antibodies (ANAs); rheumatoid factor (RF); erythrocyte sedimentation rate (ESR); and highly sensitive C-reactive protein (hsCRP) tests.

### Statistical methods

Statistical analyses were performed using the IBM SPSS Statistics version 17.0 software (IBM, Armonk, NY, USA) and the Prism software version 6.1 (GraphPad Software, La Jolla, CA, USA). The data are reported as the means ± SD or median (Q1–Q3). The normally distributed data between two groups were analysed using independent-samples *t* tests or paired-samples *t* tests, and a one-way analysis of variance (ANOVA) was used to compare the groups. Categorical data were analysed using the chi-square test, while the non-normally distributed data were analysed using the rank sum test. A two-tailed *P* value < 0.05 was considered significant.

## Results

### Clinical characteristics of the IgG4-RD patients

A total of 121 newly diagnosed IgG4-RD patients were enrolled in our study. According to the diagnostic criteria, 74 (61.2%), 44 (36.4%), and 3 (2.5%) of patients were definite, possible, and probable IgG4-RD, respectively. The number of patients in groups A, B, and C was 34, 41, and 46. The demographic features are listed in Table 1. The average ages in groups A, B, and C were 49.4 ± 14.0, 53.8 ± 16.7, and 55.5 ± 11.1 years, and there were no statistically significant differences among the three groups. Disease duration of group A to group C was 21.5 (IQR 12–51) months, 6 (IQR 2–12) months, and 21.5 (IQR 6–51) months. The disease duration in groups A and C was longer than that in group B (*P* < 0.001). The biopsy rate of groups A (70.6%) and C (84.8%) was higher than that of group B (36.7%) (*P* < 0.001). More notably, groups A (61.8%) and C (69.6%) had a higher prevalence of allergies than group B (*P* = 0.003) (Table 1), and the most common allergic symptoms of patients with IgG4-RD were allergic rhinitis and asthma. The number of organs affected in three groups was 3.3 ± 1.1, 2.6 ± 1.1, and 5.3 ± 1.5. The IgG4-RD RI was 11.5 (Q1–Q3, 9.0–14.0), 11.0 (Q1–Q3, 7.5–15.0), and 19.0 (Q1–Q3, 15.0–24.0).

**Table 1:** Clinical characteristics of 121 IgG4-RD patients with and without DS

Variables	Group A (*n* = 34)	Group B (*n* = 41)	Group C (*n* = 46)	*P* value
Age (years)	49.4 ± 14.0	53.8 ± 16.7	55.5 ± 11.1	0.155
Sex(male/female)	1.83/1	4.13/1	2.29/1	0.288
Disease duration (months), M (Q1–Q3)	21.5 (12–51)	6 (2–12)	21.5 (6–51)	< 0.001^#^
Biopsy (*n*, %)	24 (70.6)	15 (36.7)	39 (84.8)	< 0.001^#^
History of allergy (*n*, %)	21 (61.8)	14 (34.1)	32 (69.6)	0.003^#^
IgG4-RD RI, M (Q1–Q3)	11.5 (9–14)	11 (7.5–15)	19.2 ± 5.2	0.0001^#^
Number of organs affected	3.3 ± 1.1	2.6 ± 1.1	5.3 ± 1.5	0.0001^#^

*P* ≤ 0.05 was considered statistically significant

*M (Q1–Q3)* median (interquartile range), *IgG4-RD RI* IgG4-RD responder index

### Symptoms at disease onset

**Fig. 1:**
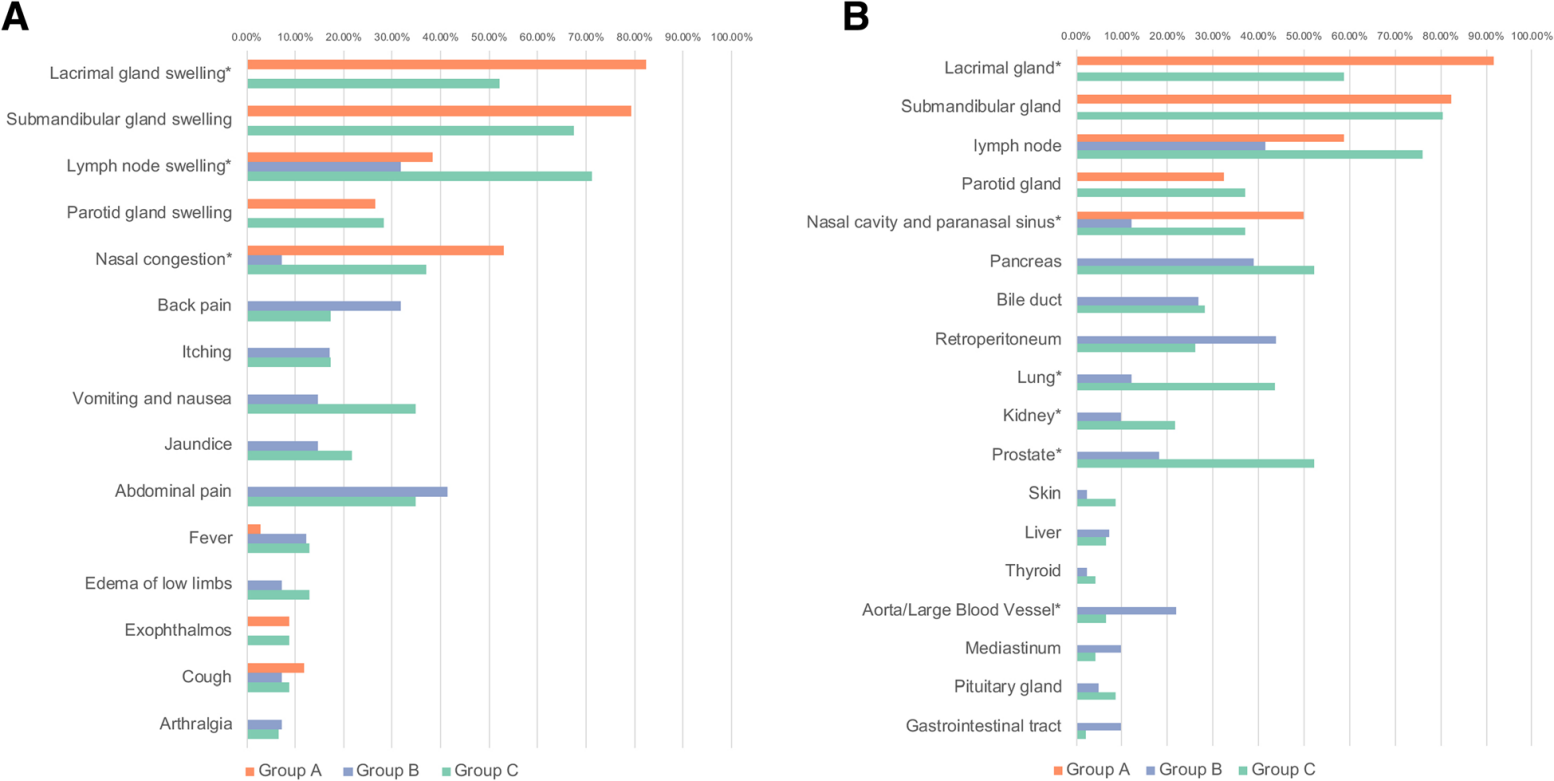
Symptoms of disease onset and affected organs in 121 IgG4-RD patients with and without DS. **a** The percentage of symptoms at disease onset. **b** The percentage of affected organs in IgG4-RD patients with and without DS at baseline. Symptom or organ with asterisk presents a statistical significance between groups

The symptoms at disease onset are shown in Fig. 1a. Lacrimal gland swelling (52, 43.0%), submandibular gland swelling (58, 47.9%), lymph node swelling (59, 48.8%), nasal congestion (38, 31.4%), abdominal pain (33, 27.3%), vomiting and nausea (22, 18.2%), and jaundice (22, 18.2%) were the common onset symptoms. More patients with DS than without DS experienced nasal congestion and/or anosmia, the percentage of nasal congestion in group A (52.9%) and group C (37%) was higher than that in group B (7.3%), *P* < 0.001. Additionally, there were more patients who presented with lacrimal gland swelling in group A (82.4%) than in group C (25.2%), *P* = 0.005. In group C, more patients had lymph node swelling (33, 71.3%) than in other two groups (group A was 13, 38.4%, and group B was 13, 31.7%), *P* < 0.001.

### Organs affected at baseline

The organs involved at baseline are shown in Fig. 1b. Consistent with the onset symptoms, the most common organs affected in our study were the submandibular gland (65, 53.7%), lacrimal gland (58, 47.9%), lymph node (72, 59.5%), pancreas (40, 33.1%), nasal cavity and paranasal sinus (39, 32.2%), and retroperitoneum (30, 24.8%). In male patients, prostatitis was also a common feature. By comparison, among the patients with DS, more in group A (91.2%) had an affected lacrimal gland than in group C (58.7%), *P* = 0.001; in addition, the percentage of patients with an affected nasal cavity and paranasal sinus in groups A (50.0%) and C (37.0%) was higher than that in group B (12.2%), *P* = 0.002. There were higher percentages of patients with lung, prostate, or kidney involvement in group C (20, 43.5%; 14, 43.8%; and 10, 21.7%, respectively) than in group B (5, 12.2%; 6, 18.2%; and 4, 9.8%, respectively), with *P* = 0.001, *P* = 0.026, and *P* < 0.001, respectively. However, there were more patients with an affected aorta/large blood vessel in group B (22%) than in group C (6.5%), *P* = 0.037.

### Laboratory parameters

Baseline laboratory parameters and results are shown in Table 2. Statistical significances were seen in the level of eosinophil, serum IgG4, IgG, ESR, and hsCRP among the three groups. The patients in group A had higher haemoglobin values than in group B, *P* = 0.016. Patients in group A [8110 (4395–15,025) mg/L] and C [15,000 (8782–27,850) mg/L] showed higher serum IgG4 levels than in group B [3240 (1870–6455) mg/L], *P* < 0.001. Similarly, group C (25.19 ± 11.7 g/L) had higher serum IgG levels than did group A (19.83 ± 7.34 g/L) and group B (18.06 ± 8.16 g/L), *P* = 0.002. Consistent with this result, more patients in group C (34, 73.9%) had an elevated serum IgG level compared with group A (17, 53.1%) and group B (19, 47.5%), *P* = 0.032. In addition, more patients presented eosinophilia in group A (32.4%) and group C (41.3%) than in group B (17.1%), *P* = 0.048. ESR was higher in group B [36(15–71)mm/h] and group C [31(13–69)mm/h] than in group A [13(5–22)mm/h], *P* = 0.002. Besides, hsCRP was also higher in group B [5.67(2.03–17.7)mg/L] and group C [2.02(0.94–7.9) mg/L] than in group A [1.28(0.55–2.18) mg/L], *P* = 0.019. In addition, the percentages of patients with an elevated ESR and hsCRP level in groups B and C were higher than those in group A, *P* = 0.007 and *P* = 0.002, respectively. Our study showed that there were no statistically significant differences in the WBC, PLT, IgA, IgM, IgG1, IgG2, IgG3, T-IgE, ALT, creatinine, ANAs, or RF results among the three groups.

**Table 2:** Laboratory parameters of 121 IgG4-RD patients with and without DS

	Group A (*n* = 34)	Group B (*n* = 41)	Group C (*n* = 46)	*P* value
HGB (g/L)	142 ± 14	130 ± 19	134 ± 18.3	0.016^#^
WBC (10^9^/L)	7.27 ± 2.81	7.72 ± 2.47	7.5 ± 3.18	0.793
PLT (10^9^/L)	237 ± 50	259 ± 105	245.7 ± 171.3	0.747
Elevated Eos% (%)	33.3 (11/33)	17.9 (7/39)	43.5	0.042^#^
ESR (mm/h), M (Q1–Q3)	13 (5–22)	36 (15–71)	31 (13–69)	0.002^#^
Elevated ESR (%)	37.5 (12/32)	73.7 (28/38)	63.6 (28/44)	0.007^#^
hsCRP (mg/L)	1.28 (0.55–2.18)	5.67 (2.03–17.7)	2.02 (0.94–7.9)	0.019^#^
Elevated hsCRP (%)	12.5 (3/24)	57.1 (20/35)	37.5 (15/40)	0.002^#^
IgG (g/L)	19.83 ± 7.34	18.06 ± 8.16	25.19 ± 11.7	0.002^#^
Elevated IgG (%)	53.1 (17/32)	47.5 (19/40)	73.9	0.032^#^
IgA (g/L)	1.84 ± 0.65	2.61 ± 1.33	1.92 ± 1.48	0.014^#^
Elevated IgA (%)	0	12.5 (5/40)	2.2	–
IgM (g/L)	1.02 ± 1.12	1.13 ± 0.83	1.02 ± 2.56	0.944
Elevated IgM (%)	9.4 (3/32)	10 (4/40)	2 (4.3)	0.560
IgG1 (mg/L), M (Q1–Q3)	7530 (6500–10,028)	9250 (7950–11,100)	8580 (6770–10,100)	0.116
Elevated IgG1 (%)	5.9 (2/34)	17.9 (7/39)	13.3 (6/45)	0.300
IgG2 (mg/L), M (Q1–Q3)	5315 (4195–7488)	5700 (4170–7110)	5390 (3455–7020)	0.679
Elevated IgG2 (%)	38.2	38.5 (15/39)	31.1 (14/45)	0.727
IgG3 (mg/L), M (Q1–Q3)	474 (260–1123)	408 (247–932)	529 (276–884)	0.843
Elevated IgG3 (%)	26.5	20.5 (8/39)	15.6 (7/45)	0.490
IgG4 (mg/L), M (Q1–Q3)	8110 (4395–15,025)	3240 (1870–6455)	15,000 (8782–27,850)	< 0.001^#^
Elevated IgG4 (*n*, %)	32 (94.1)	36 (87.8)	46 (100)	–
T-IgE (KU/L), M (Q1–Q3)	338 (76.6–555.3)	236 (102–686)	399 (169–1040.8)	0.447
Elevated IgE (%)	82.1 (23/28)	90.3 (28/31)	95 (38/40)	0.222
ALT (u/L)	31 ± 43.9	44.6 ± 89.9	39.8 ± 82.2	0.763
Cr (μmol/L)	67.3 ± 14.7	96.4 ± 89.8	78.3 ± 36.5	0.088
ANA+ (*n*, %)	4 (11.8)	3 (7.3)	8 (17.4)	0.360
RF+ (*n*, %)	8 (23.5)	5 (12.2)	3 (6.5)	0.083

*Cr* creatinine, *ANA* anti-nuclear antibody, *RF* rheumatoid factor

^#^There was a statistical significance

### Treatment and efficacy

**Fig. 2:**
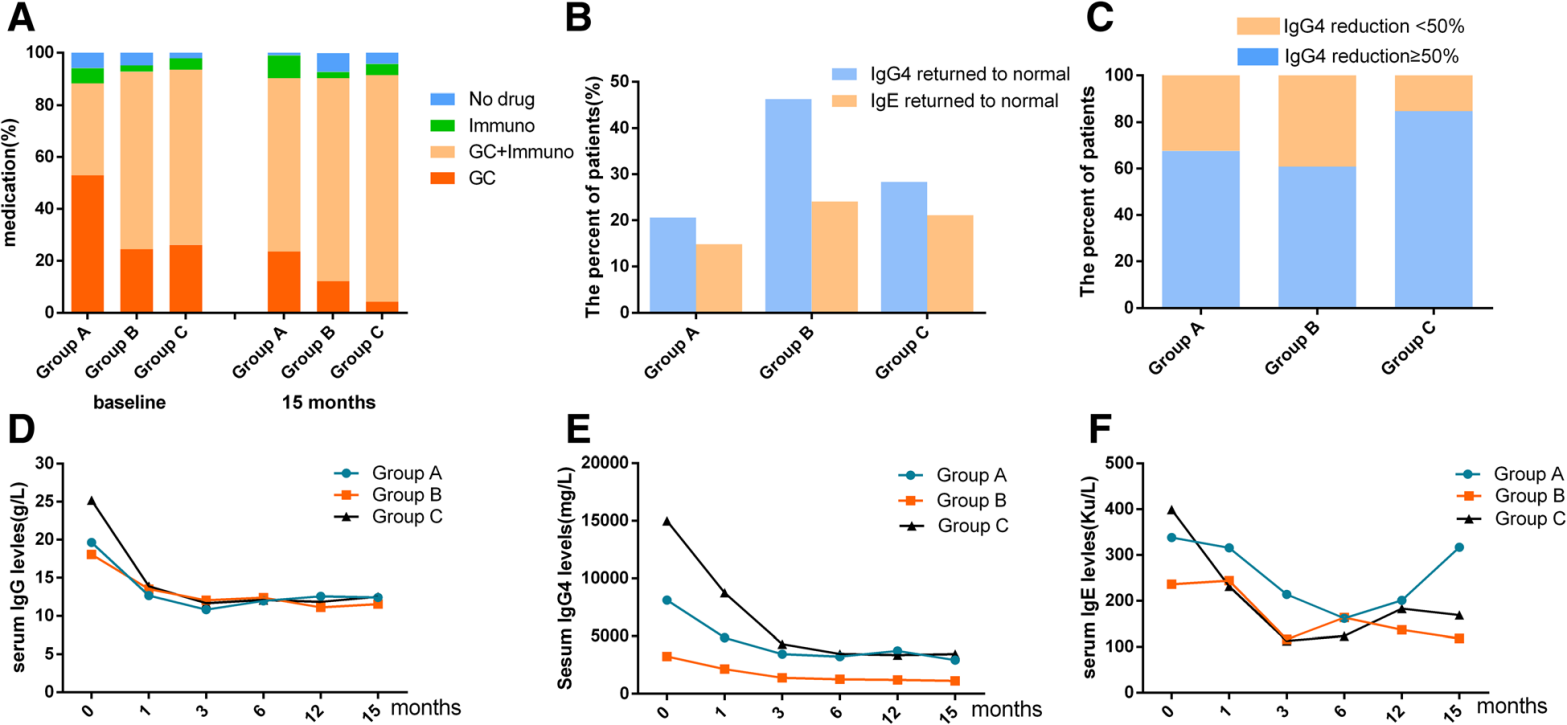
Medications and laboratory parameters of patients with and without DS. **a** The percentage of patients’ drug use of each group. **b** The percentage of patients whose serum IgG4 and IgE returned to normal. **c** The percentage of patients whose serum IgG4 reduction more than 50%. **d–f** The serum IgG4, IgG, and IgE levels reduction after treatment

Altogether, 91.7% of the patients were treated with glucocorticoids at diagnosis. The initial doses of prednisone/prednisolone were 20 to 50 mg/day, and the majority of patients were given 30 to 40 mg/day (0.5–0.6 mg/kg/day) [23, 24]. In groups A, B, and C at baseline, there were 18 (52.9%), 10 (24.4%), and 12 (26.1%) patients, respectively, treated with glucocorticoid monotherapy; there were 12 (35.3%), 28 (68.3%), and 31 (67.4%) patients treated with glucocorticoids combined with immunosuppressants in groups A, B, and C, respectively (Fig. 2a). The most common immunosuppressant used in our study was cyclophosphamide and mycophenolate for patients with internal organ involvement (groups B and C) and azathioprine and methotrexate for the DS-predominant patients (group A). Other immunosuppressants included *Tripterygium wilfordii*, cyclosporin, and tamoxifen. The number of patients treated with immunosuppressive monotherapy was 2 (5.9%), 1 (2.4%), and 2 (4.3%) in groups A, B, and C. Only 2 (5.9%), 2 (4.9%), and 2 (4.35%) patients in groups A, B, and C, respectively, received wait-and-see treatment. At baseline, a higher percentage of group A was on glucocorticoids compared with group B and group C (*P* = 0.011, *P* = 0.014, respectively). The treatment of glucocorticoids combined with immunosuppressant was received by more patients in groups B and C than in group A (*P* = 0.005). Most of the relapsed patients were given additional immunosuppressants; therefore, at the 15-month follow-up, the proportion of patients on combination treatment increased (Fig. 2a).

All the patients responded well to the treatments, and the IgG4-RD RI decreased significantly after the treatments; 1 patient in group A, 3 patients in group B, and 2 patients in group C were able to stop medication. At the 15-month follow-up, the IgG4-RD RI values of the patients in groups A, B, and C were 2.53 ± 2.44, 1.56 ± 1.78, and 1.74 ± 1.08, respectively. At the 15-month follow-up, the serum IgG (43.8% in group A, 40.0% in group B, and 60.9% in group C), IgG4 (20.6% in group A, 46.3% in group B, and 28.3% in group C), and IgE (14.8% in group A, 24.1% in group B, and 21.1% in group C) levels were able to return to normal in part of our patients (Fig. 2b, Table 3). In more than 60% of the patients, the reductions in the serum IgG, IgG4, and IgE were ≥ 50% (Fig. 2c, Table 3).There was a significant reduction in the levels of serum IgG, IgG4, and IgE at the 1st month and 3rd month (Fig. 2d–f). Unlike IgG and IgG4, there was an increase in the level of serum IgE of the patients at the 6th month(Fig. 2f). In total, 28 (23.1%) patients had a disease relapse during the 15 months of follow-up: 8 patients had lacrimal gland relapse, 5 patients had a nasal cavity and paranasal sinus relapse, 4 patients had submandibular re-swelling, 4 patients had a pancreatic relapse, 2 patients had a lung relapse, 1 patient had kidney disease relapse with increased urine protein, 1 patient had retroperitoneal fibrosis re-enlargement, 1 patient had parotid gland re-swelling, and 1 had lymph node relapse. The number of patients who relapsed from groups A, B, and C was 11 (32.3%), 8 (19.5%), and 8 (17.4%), respectively. All patients had 1 organ relapse with the exception that 2 patients in group A had 2 organs affected.

**Table 3:** Laboratory parameters at 15 months of follow-up compared with the baseline

	Group A (*n* = 34)	Group B (*n* = 41)	Group C (*n* = 46)	*P* value
Serum IgG at 15 months (g/L)	12.23 ± 3.88	11.32 ± 4.20	12.26 ± 4.48	0.568
IgG returned to normal (%)	43.8 (14/32)	40 (16/40)	60.9	0.120
IgG reduction ≥ 50% (%)	31.3 (10/32)	27.5 (11/40)	25 (54.3)	0.023^#^
Serum IgG4 at 15 months (mg/L), M (Q1–Q3)	2730 (1199–4768)	1140 (679–2795)	3445 (1004–8340)	< 0.001^#^
IgG4 returned to normal (*n*, %)	7 (20.6)	19 (46.3)	13 (28.3)	0.035^#^
IgG4 reduction ≥ 50% (*n*,%)	23 (67.6)	28 (60.9)	39 (84.8)	0.121
Serum IgE of 15 months (KU/L), M (Q1–Q3)	218 (68.5–496.5)	118 (30.8–427)	163.5 (38.9–428.8)	0.201
IgE returned to normal (%)	14.8 (4/27)	24.1 (7/29)	21.1 (8/38)	0.677
IgE reduction ≥ 50% (%)	29.6 (8/27)	44.8 (13/29)	57.9 (22/38)	0.078

#There was a statistical significance

## Discussion

IgG4-RD is a novel clinical entity with multi-organ involvement and variable clinical manifestations. DS patients with elevated levels of serum IgG4 are recognized as a subset of IgG4-RD [25]. To clarify the differences and similarities between DS as a subgroup of IgG4-RD and other IgG4-RD subtypes, we compared the clinical manifestations and treatment responses in IgG4-RD patients with or without DS from a large cohort in China.

In this study, we found that patients with DS had longer disease durations than those without. Allergies and nasal sinus lesions were more common in patients with DS. In addition, patients with DS had a higher percentage of eosinophilia and higher levels of serum IgG, IgG4, and IgE than those without DS. The serum IgG and IgG4 levels were the highest in group C. Compared with those in groups A and C, the serum IgG4 levels were lower in group B and more readily returned to normal after treatment. Elevated serum IgG4 is not specific to IgG4-RD and can be found in many other diseases, such as malignancy, systemic vasculitis, infectious diseases, and other conditions [6]. Malignancies such as lymphoma sometimes mimic IgG4-RD, and therefore, confirmation via biopsy is highly recommended for differential diagnosis [26–28]. A biopsy of superficial organs, such as the lacrimal or submandibular glands, is convenient and therefore more advantageous. In our patients, the biopsy rate in groups A and C was higher than that in group B.

In addition to swelling of the lacrimal and salivary glands, many IgG4-RD patients with DS also had nasal congestion. The patients in groups A and C were more likely to have nasal symptoms than those in group B (Fig. 1). Rhinosinusitis is common in IgG4-RD patients, especially in DS patients [29]. It has been reported that 9 of 13 DS patients have chronic rhinosinusitis (CRS), and 41 of 79 IgG4-RD patients exhibited CRS in another study [29, 30]. Consistent with the study of Della Torre et al., allergic rhinitis and asthma were common in IgG4-RD, but the percentage of patients with a history of allergy in our study was higher than that in the study of Della Torre et al. [31]. Although IgG4-RD patients with CRS were thought to represent a new clinical entity of nasal disease, its pathogenesis can be similar to eosinophilic CRS presented as an IgG4+ plasma infiltration into the nasal mucosa. However, there were no significant differences in the nasal mucosal eosinophil infiltration and the IgG4-positive plasma cell/IgG-positive plasma cell ratios compared with common CRS [30]. With elevated IgG4 levels and other evidence, the nasal cavity and sinus lesions suggesting CRS may be only one of the extra-glandular symptoms of DS but are not possible diagnostic lesions.

Many IgG4-RD patients have been reported to have features of atopic disease [5]. Stone et al. estimated that up to 40% of these patients might have evidence of atopic disease [32]. The common atopic symptoms in our groups were allergic rhinitis, asthma, and atopic dermatitis, which is similar to the findings of Della Torre et al., and the percentage with a history of allergy was even higher, especially in those with DS [31]. The prevalence of elevated serum IgE is higher in bronchial asthma and allergic rhinitis than in asthma [29]. The level of serum IgE was increased in over 80% of our patients, and there were no differences among the three groups. In a Japanese cohort, eosinophilia and high levels of serum IgE and IgG4 are common features regardless of an allergy; with or without an allergy, the response to steroids and the proportion of patients who relapsed were not significantly different [15]. Nasal congestion was more commonly seen in patients with DS (groups A and C), who had a higher serum IgE level than group B at both the baseline and the 15-month follow-up. Consistent with a previous report [33], the IgE and IgG4 levels began to decrease with a decrease in IgG4 after treatment (Fig. 2f, i), and nasal symptoms were alleviated. However, our data showed an ascending trend of IgE from the 6th to the 15th month in group A, and an up-down wave was also seen in the 3rd to the 12th month in group B and in the 6th to the 15th month in group C (Fig. 2f), although the serum IgG4 level and disease status appeared stable. The baseline IgE is a relapse risk factor [33]. It has been reported that atypical manifestations of asthma or sinusitis are ameliorated after the application of steroids, but serum IgE levels do not reflect disease activity in asthma or allergic rhinitis [34]. In this study, many of our patients had a reduction in IgE ≥ 50%, but only a small portion of the patients had IgE levels that returned to normal. Also, IgE in group A in the 15th month elevated without statistically changing the IgG4 level.

Generally, glucocorticoids are still the first-line treatment for IgG4-RD. In this study, most of the patients were treated with glucocorticoids alone or combined with immunosuppressants, according to the disease severity. Patients with internal organ involvement (groups B and C) were treated more aggressively than those without (group A). The most commonly used immunosuppressant in our study was cyclophosphamide and mycophenolate mofetil for patients with internal organ involvement, while azathioprine and methotrexate for DS-predominant patients. The IgG4-RD RI decreased significantly after treatment. Steroid treatment can lead to a rapid remission in salivary gland swelling and in salivary secretion, while improving hypergammaglobulinemia [35, 36]. In our study, all 121 patients responded to therapy, and 6 (5.0%) patients were able to stop medication.

Disease relapse frequently occurs in organs other than the pancreas following an initial course of steroids [4, 37]. In IgG4-RD, there have been several reports regarding the relapse of DS patients. In a Japanese cohort, male sex and a younger onset were predictors of relapse in the group without organ lesion other than the lacrimal and salivary glands in IgG4-related dacryoadenitis and sialoadenitis [38]. High baseline serum IgG4 levels before treatment and a lower dose and shorter duration of initial steroid treatment have been associated with recurrence [39]. Our study showed that 28 (23.1%) patients had disease relapse during the 15 months of follow-up, and thus, most of our patients added extra immunosuppressants to prevent relapse. The relapse rate in our study is consistent with that of another report in which the relapse rate was 20% in patients receiving maintenance therapy [40] which is similar to the results of Kamisawa et al., who reported a relapse rate of 23% in glucocorticoid maintenance therapy [22]. However, the rate in our study was lower than the rates of 32.5% and 43% reported by two other centres [36, 39]. Nevertheless, after steroids were discontinued, the swelling of the glands recurred along with an increase in serum IgG4 levels. The most common site of relapse in our study was the lacrimal gland, followed by the nasal sinus and pancreas. A serologically unstable condition has been observed to occur in 54.9% of IgG4-related sialoadenitis patients, who then received treatment adjustment [36]. Additionally, patient with recurring IgG4-related ophthalmic disease eventually required significantly more steroids than did those without a recurrence [39]. Therefore, this scenario requires prolonged steroid treatment or a combination of steroids and an immunosuppressant [12].

There are some limitations to this study. First, the number of patients in this study is relatively small because the disease of interest is rare. Second, the follow-up duration was not long. Third, although more than 50% of the patients had a history of allergies, some of the patients in our study did not go through allergen detection process, or with actual allergens remained unclear. Fourth, because this was a real world study, patients in the three groups were treated differently; we could not make conclusions of treatment efficacy and prognosis among different groups.

## Conclusion

Our study demonstrated that IgG4-RD patients with DS were affected by a higher percentage of allergy and sinonasal conditions; the serum IgG4 levels were higher, which may suggest a different mechanism of pathogenesis from that occurring in IgG4-RD patients without DS.

## Additional file

Additional file 1: Final RI Scoring Sheet of IgG4-RD Responder Index Validation Study. (PDF 140 kb)

## Abbreviations

IgG4-RD: IgG4-related disease
MD: Mikulicz’s disease
DS: Dacryoadenitis and sialoadenitis
SS: Sjogren’s syndrome
GC: Glucocorticoids
ESR: Erythrocyte sedimentation rate
hsCRP: Highly sensitive C-reactive protein
ANAs: Anti-nuclear antibodies
RF: Rheumatoid factor
Cr: Creatinine
WBC: White blood cell
PLT: Platelets
T-IgE: Total IgE
CRS: Chronic rhinosinusitis
RI: Responder index
OR: Odds ratio
