# Supplementary material for: Differences and similarities between IgG4-related disease with and without dacryoadenitis and sialoadenitis: clinical manifestations and treatment efficacy
Source: Arthritis Res Ther. 2019 Feb 1;21:44. doi: 10.1186/s13075-019-1828-8 (PMC6359790; doi:10.1186/s13075-019-1828-8)
Supplement: Supplementary file 1 — Final RI Scoring Sheet of IgG4-RD Responder Index Validation Study. (PDF 140 kb) [file 13075_2019_1828_MOESM1_ESM.pdf]

IgG4-RD Responder Index Validation Study

Scoring Rules
Scoring refers to manifestations of disease activity present in the last 28 days

Scoring: 0 Normal or resolved
1 Improved but still present
2 Persistent (still active; unchanged from previous visit)
3 New / Recurrence while patient is off treatment
4 Worsened or new disease manifestation despite treatment

Definitions
Organ/Site score: The overall level of IgG4-RD activity within a specific organ system
Symptomatic: Is the disease manifestation in a particular organ system symptomatic? (Y = yes; N = no)
Urgent disease: Disease that requires treatment immediately to prevent serious organ dysfunction (Y = yes; N = no)
(*Presence of **urgent disease** within an organ leads to **DOUBLING** of that organ system score*)
Damage: Organ dysfunction that has occurred as a result of IgG4-RD and is considered permanent (Y = yes; N = no)

	**Activity**			**Damage**	
Organ/Site	Organ/Site Score (0-4)	Symptomatic (Yes/No)	Urgent (Yes/No)	Yes/No	Symptomatic (Yes/No)
Meninges					
Pituitary Gland					
Orbital lesion (specify location): ______________________________					
Lacrimal Glands					
Parotid Glands					
Submandibular Glands					
Other Salivary Glands (specify): _______________________					
Mastoiditis / Middle ear disease					
Nasal Cavity Lesions					
Sinusitis					
Other ENT Lesions, e.g., tonsillitis, pharyngitis (specify): ______________________					
Thyroid					
Lungs					
Lymph Nodes (please circle site of involvement, below):					
Submental Submandibular Cervical Axillary Mediastinal Hilar Abdominal/Pelvic Inguinal Other lymph node chains:					

	Activity			Damage	
Organ/Site	Organ/Site Score (0-4)	Symptomatic (Yes/No)	Urgent (Yes/No)	Yes/No	Symptomatic (Yes/No)
Aorta / Large Blood Vessels					
Heart/Pericardium					
Retroperitoneal Fibrosis					
Sclerosing Mediastinitis					
Sclerosing Mesenteritis					
Pancreas					
Liver					
Bile ducts					
Kidney					
Skin					
Constitutional symptoms not attributable to involvement of a particular organ (weight loss, fever, fatigue caused by active IgG4-RD)					
Other involvement - specify:<br><br>(Consider prostate, breast, gallbladder involvement; and other. Each “Other” item is counted separately.)<br><br>________________<br><br>________________	________<br><br>________	________<br><br>________	________<br><br>________	________<br><br>________	________<br><br>________

Total Activity Score

Organ/sites (x 2 if urgent): ______

Total **urgent** organs: __________

Total **symptomatic (active)** organs: __________

Total **damaged** organs: __________

Total **symptomatic (damage)** organs: __________

Physician Global Assessment (PGA) of disease activity

0 ———————————————— 100 mm

PGA = ________ mm
